# Modeling of crop wild relative species identifies areas globally for in situ conservation

**DOI:** 10.1038/s42003-019-0372-z

**Published:** 2019-04-23

**Authors:** Holly Vincent, Ahmed Amri, Nora P. Castañeda-Álvarez, Hannes Dempewolf, Ehsan Dulloo, Luigi Guarino, David Hole, Chikelu Mba, Alvaro Toledo, Nigel Maxted

**Affiliations:** 1 School of Biosciences, University of Birmingham, Birmingham, B15 2TT UK; 2 International Centre for Agricultural Research in the Dry Areas, Rabat, Morocco; 3 International Center for Tropical Agriculture (CIAT), Km 17, Recta Cali-Palmira, Cali, Colombia; 4 Global Crop Diversity Trust, Platz der Vereinten Nationen 7, 53113 Bonn, Germany; 5 Bioversity International, Via dei Tre Denari 472/a, 00057 Maccarese (Fiumicino), Roma, Italy; 6 Moore Center for Science, Conservation International, Arlington, VA 22202 USA; 7 Center for Biodiversity Outcomes, Arizona State University, Tempe, AZ 85281 USA; 8 Plant Production and Protection Division, Food and Agricultural Organization (FAO), Viale delle Terme di Caracalla, 00153 Rome, Italy; 9 Secretariat of the International Treaty on Plant Genetic Resources for Food and Agriculture, FAO, Viale delle Terme di Caracalla, 00153 Rome, Italy

**Keywords:** Plant sciences, Ecology

## Abstract

The impact of climate change is causing challenges for the agricultural production and food systems. More nutritious and climate resilient crop varieties are required, but lack of available and accessible trait diversity is limiting crop improvement. Crop wild relatives (CWR) are the wild cousins of cultivated crops and a vast resource of genetic diversity for breeding new, higher yielding, climate change tolerant crop varieties, but they are under-conserved (particularly in situ), largely unavailable and therefore underutilized. Here we apply species distribution modelling, climate change projections and geographic analyses to 1261 CWR species from 167 major crop genepools to explore key geographical areas for CWR in situ conservation worldwide. We identify 150 sites where 65.7% of the CWR species identified can be conserved for future use.

## Abstract (web summary)

Holly Vincent et al. use species distribution modeling, climate change projections, and geographical analyses of more than 1200 crop wild relatives, identifying 150 key conservation areas most effective in preserving genetic diversity of crop wild relatives.

## Introduction

Ensuring global food security now and for the future is one of the greatest challenges of our time. One in nine people worldwide suffer from chronic hunger^1^, and with the human population projected to rise to 9.7 billion by 2050—meaning an extra 2.2 billion mouths to feed^2^, the pressure on food production systems is likely to increase dramatically^3^. Developing new crop varieties able to withstand climatic extremes, endure altered or increased exposure to pests and diseases, and be more resource efficient requires access to as broad a range as possible of plant genetic resources, and a far greater range than exists today^4^. Crop wild relatives (CWR), the wild and weedy plants closely related to cultivated crops, are a rich source of novel genetic diversity for crop breeding^5^. Despite their value for food and agriculture, globally CWR are poorly represented ex situ in gene banks^6^, although systematic effort to improve ex situ coverage has begun^7^. Further, only a handful of genetic reserves for active in situ conservation exist^8^, despite the generally accepted requirement for complementary conservation^9^ and the particular need to develop CWR in situ activities that enable the conservation of geographical partitioned genetic diversity which retains potential for local environmental-evolutionary adaptation^10^. Furthermore, existing in situ reserves do not meet the required management standards to maintain CWR populations and their genetic diversity long-term^11^. The most effective means of systematically ensuring in situ CWR conservation would be to establish a global network of in situ populations actively managed to maintain genetic diversity^12^. Here, we tackle the in situ CWR conservation deficit and, to our knowledge, for the first time address which sites and CWR populations might most effectively form the foundation for a global network of reserves for priority CWR in situ conservation. The selection of such sites and CWR populations needs to consider climate change resilience, maximize potential CWR taxonomic and genetic diversity inclusion and where feasible, use the existing global network of protected areas to avoid the expensive establishment of reserve sites and minimize the impact of human habitat modification associated for example with agriculture, forestry and urbanization. Addressing these challenges will contribute to achieving globally agreed goals on biodiversity and sustainable development. CWRs are explicitly mentioned in Target 13 of the Convention on Biological Diversity’s Aichi Targets^13^ and UN Sustainable Development Goal 2 – Ending Hunger, Target 2.5 “maintain genetic diversity of… cultivated plants... and their related wild species…”^14^.

## Results

### Modeling global CWR richness

We identified a total of 1425 CWR species, related to 167 crops, as priority CWR for improving food security and income generation (supplementary data 1). Some CWR species belong to more than one crop genepool, for example, *Brassica cretica* Lam. belongs to the secondary genepool of both kale and oil seed rape. A total of 164 species (of the 1425 species—11.5%) had no occurrence records, leaving a total of 1261 CWR species related to 167 crops to analyze. In total, we gathered 136,576 CWR occurrence records with unique coordinates. We modelled the distributions of 791 CWR using MaxEnt, but 67 of these models did not meet our model adequacy criteria. We therefore produced a circular buffer of 50 km around occurrence records for such cases and for the remaining 537 CWR that had fewer than 10 occurrence records to produce an adequate distribution model.

**Fig. 1:**
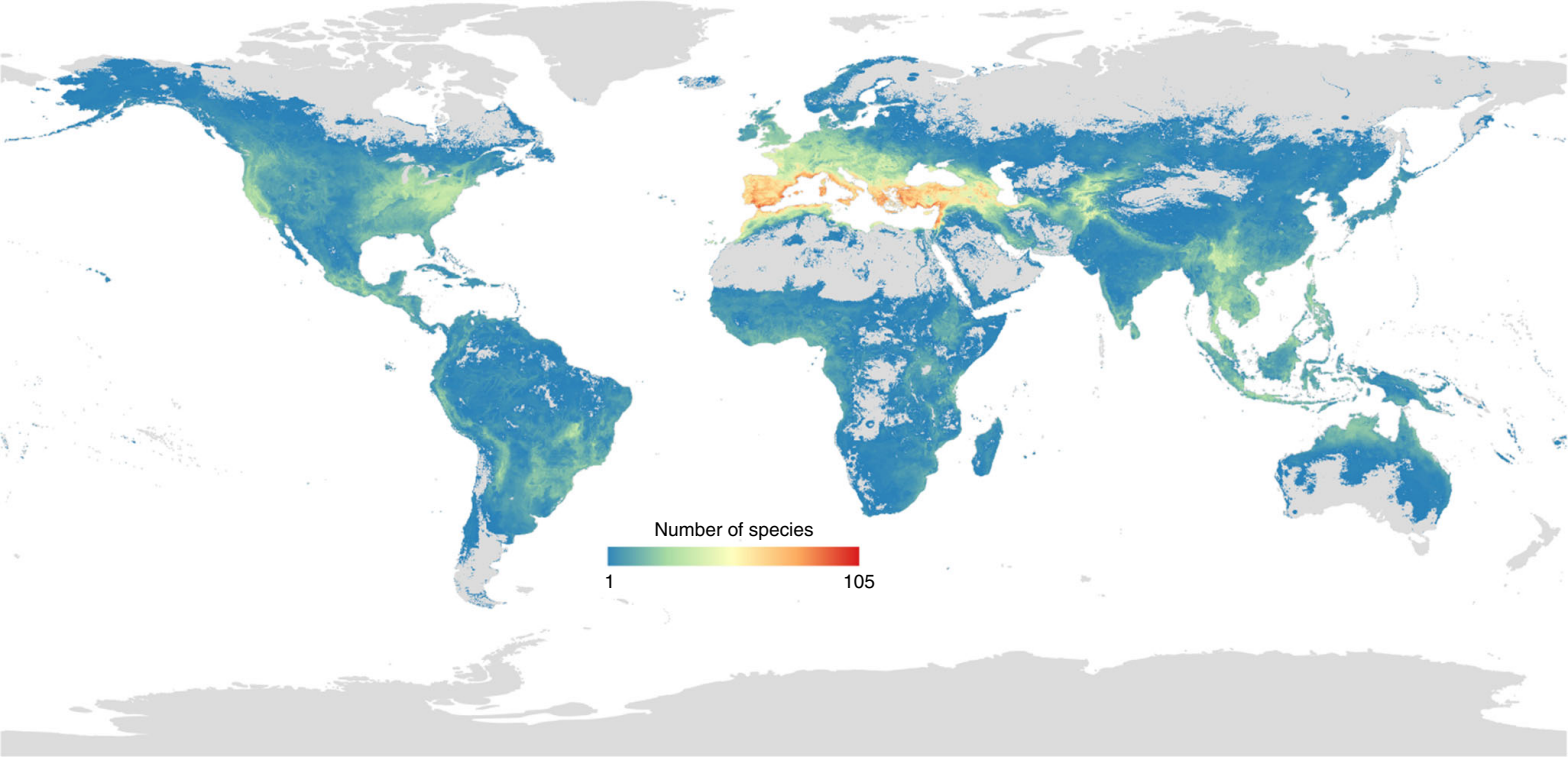
CWR species richness map. This map shows the overlapping distributions of 1261 species related to 167 crops in the world. Orange to red colours indicate high CWR species overlap, while blue to green colours indicate low overlap of CWR

Current CWR distributions are predicted to occur across most of the temperate, tropical and subtropical regions (excluding polar and extreme arid areas) (Fig. 1). CWR species are concentrated in the Mediterranean basin, previously identified as a global hotspot, with the highest concentration globally predicted to occur in a single 100 km^2^ cell on the northeast Lebanese/Syrian border^15^. Other areas of species richness include the Caucasus, Indochina, eastern USA, western coast of USA, the Andes and central and eastern South America, confirming previous species richness patterns^6^. Regions of high CWR species richness are largely coincident with areas of biodiversity richness^16^, particularly in Indochina, western coastal USA, the Andes and the Mediterranean.

### Modeling in situ gap analysis

Table 1 summarizes the in situ gap analysis results for each crop genepool, summarized by crop types^17^. Numbers of CWR species per crop type ranged from 15 for citrus fruits to 264 for root, bulb, or tuberous vegetables, which contains crops with large genepools, such as potato and cassava. The number of CWR projected to lose 50% or more of their current ranges by 2070 under 726 CWR/adaptive climate change scenarios were totaled for each crop type; the root, bulb, or tuberous vegetables have the most CWR facing potential substantial distribution loss, with 20 CWR facing over 50% current range loss, followed by cereals with 19 and leguminous crops with 17 CWR. No modelled CWR from grape crops or citrus fruits were found to lose more than 50% of their current distribution. Of CWR that are set to lose more than 50% of their current potential substantial distribution, those of spice crops are the most vulnerable, with 26.7% of all modelled CWR losing distribution by 2070, followed by sugar CWR (14.3%), cereals CWR (13.7%) and beverages (13.6%). Under the consolidated crop types, CWR are not well covered by the existing global protected area network, with grape CWR having the least coverage at 14.7% and CWR of leafy or stem vegetables having the most protected area coverage at 32.8% on average (Table 1). However, the results for loss of current distribution by 2070 show that most crops will be impacted by climate change, losing ~20% of current protected area coverage on average per CWR. The crops least affected appear to be citrus fruits, with only 4.6% loss, and the most affected being sugar crops with 31.4%.

**Table 1:** Consolidated in situ gap analysis results for different crop types

Crop type	No. crops	No. CWR	No. CWR with no occurrences	No. CWR with 1–9 occurrences	No. CWR with >50% distribution loss in 2070	Average current protected area cover for CWR (%)	Average loss of protected area cover for CWR in 2070 (%)
Berries	4	55	5	12	1	30.54	15.70
Beverage crops	5	69	26	21	3	25.56	24.98
Cereals	16	157	5	13	19	21.63	25.39
Citrus fruits	7	15	7	7	0	18.79	4.57
Fruit-bearing vegetables	10	42	3	11	2	17.60	23.23
Grapes	3	20	2	5	0	14.66	20.33
Leafy or stem vegetables	15	89	11	29	4	32.84	23.89
Leguminous crops	30	208	10	52	17	22.67	21.89
Nuts	8	73	8	29	3	24.06	19.98
Oilseed crops	11	81	7	11	4	22.50	19.47
Pome and stone fruits	10	128	19	42	3	24.27	21.97
Root, bulb, or tuberous vegetables	20	264	24	77	20	21.74	22.13
Spice crops	14	31	8	8	4	27.27	24.16
Sugar crops	2	20	1	5	2	30.27	31.37
Tropical and subtropical fruits	10	172	34	58	7	19.27	23.35
Other crops (e.g. fibres)	2	36	2	21	1	22.78	28.89

**Fig. 2:**
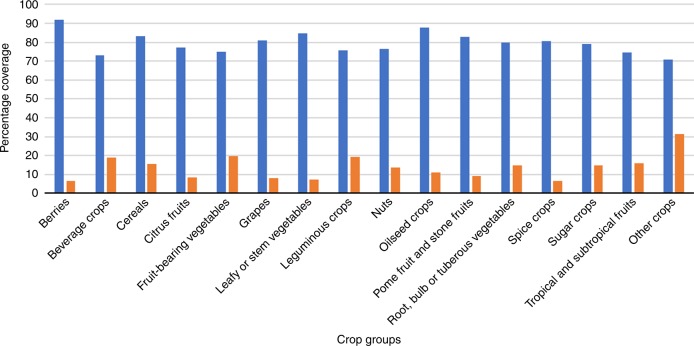
Current and projected loss of potential genetic diversity in protected areas for CWR grouped by crop type. Blue bars indicate average current coverage of genetic diversity per CWR in protected area and magenta bar indicates average loss of genetic diversity per CWR in protected area in 2070

The current proportion of potential CWR genetic diversity based on Ecogeographic Land Characterization (ELC) diversity within the existing protected areas was recorded for each species, then summarized under each crop type. Figure 2 highlights the average proportion of potential CWR genetic diversity covered by existing protected areas and the predicted losses of genetic diversity within these areas under projected climatic changes in 2070. CWR of all crop types have at least 70% of averaged potential CWR genetic diversity within the existing protected areas, with the highest being 91.9% for berries and the lowest 70.7% for other crops categories^17^. In terms of predicted loss of genetic diversity in protected areas, berries and spice crops are expected to experience the least loss, with only 6.5% reduction of genetic diversity, whilst other crops are expected to lose 31.2% of genetic diversity within protected areas, followed by fruit-bearing vegetables at 19.8% and leguminous crops at 19.5%.

Individual CWR in general were found to be well represented in current protected areas; only 35 (2.5%) of the studied species related to 28 crops were distributed exclusively outside of protected areas (supplementary data 1). These included seven CWR from primary genepools, such as wild *Pennisetum glaucum* (L.) R.Br., related to pearl millet; *Prunus argentea* (Lam.) Rehder, related to almond, and *Prunus sibirica* L., related to apricot. The top five CWR found to have the highest proportion of distribution in protected areas were: *Coffea costatifructa* Bridson related to coffee, *Ficus glareosa* Elmer related to fig, *Manihot alutacea* D.J. Rogers & Appan related to cassava, *Beta patula* Aiton and *Beta nana* Boiss. & Heldr. Both related to beet. If a threshold of 50% or more of CWR genetic diversity within protected areas is considered adequate for genetic conservation, then 112 of the assessed CWR are under-conserved and 91% of CWR are well represented by existing protected areas. However, this existing in situ conservation is likely to be passive, meaning that currently CWR populations located in protected areas are not being actively managed and monitored to maintain their diversity; more active conservation is recommended for these populations to ensure their genetic diversity is conserved^18^.

In terms of future climate projections, only two of the 724 modelled CWR species—*Vicia hyaeniscyamus* Mouterde and *Zea perennis* (Hitchc.) Reeves & Mangelsd.—are likely to lose all their current predicted distribution by 2070. However, a further 83 species are predicted to lose more than 50% of their current range by 2070 and *Arachis batizocoi* Krapov. & W. C. Greg., *Arachis appressipila* Krapov. & W. C. Greg., *Manihot gabrielensis* Allem, *Vigna keraudrenii* Du Puy & Labat and *Oryza nivara* S. D. Sharma & Shastry are all predicted to lose over 80% of their current potential distribution. Regarding potential CWR genetic diversity in 2070 based on ELC zonation, 15 CWR are projected to lose over 50% of their current genetic diversity by 2070 through distribution loss and 39 CWR are expected to lose over 50% of their genetic diversity that is currently passively conserved in protected areas. Further details on the in situ gap analysis results for individual CWR are available (supplementary data 1).

**Fig. 3:**
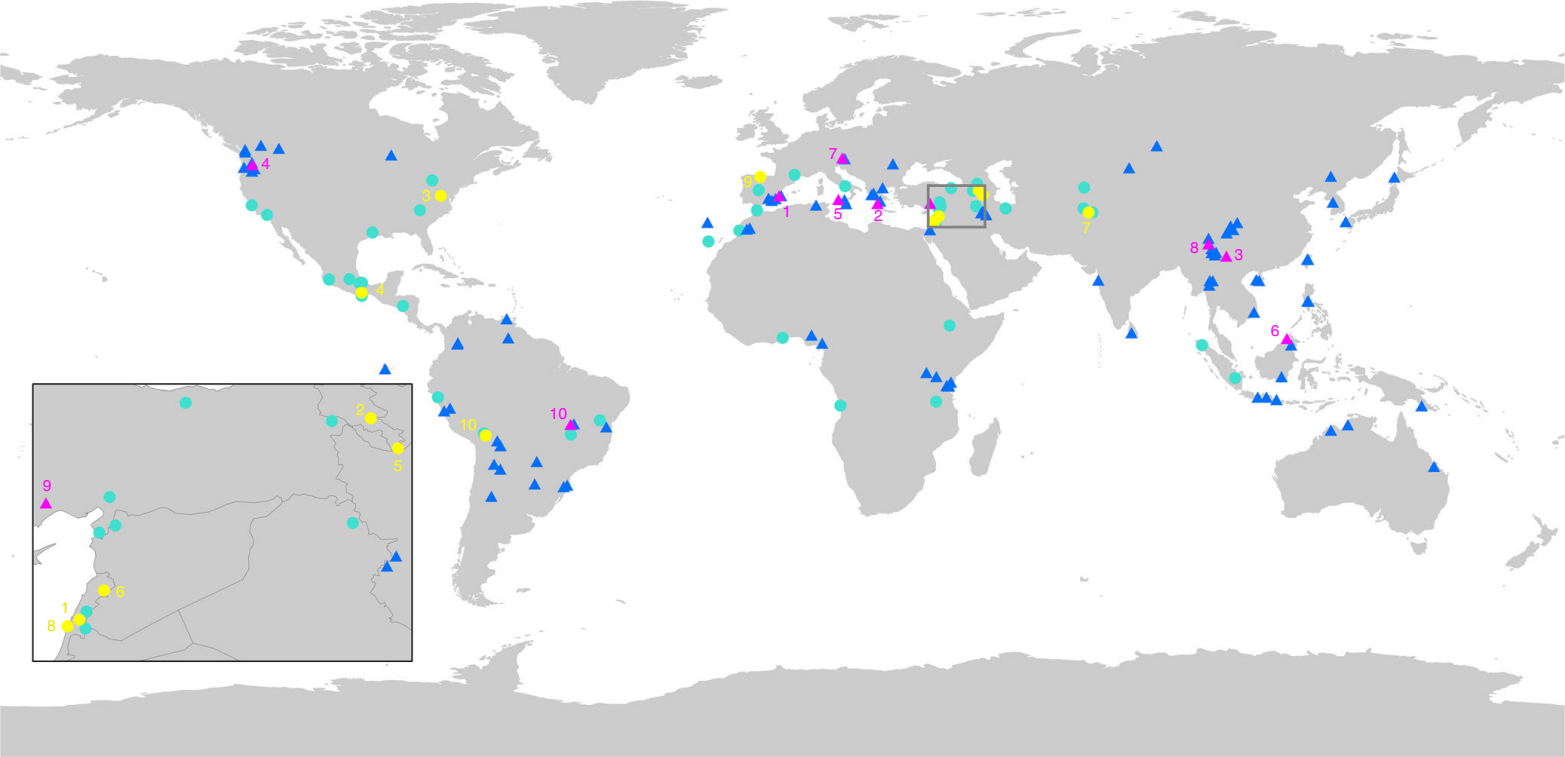
Top 150 global sites for CWR in situ conservation under the pragmatic scenario, with the enclosed map shows the priority sites in the Fertile Crescent and Caucasus. The top 10 sites within existing protected areas are shown in magenta triangles, the remaining 90 priority sites within protected areas are in blue triangles; the top 10 sites outside of existing protected areas are in yellow circles, with the remaining priority 40 sites outside of protected areas in turquoise circles

While when identifying sites for global in situ conservation of CWR, the top 150 sites (Fig. 3) covering 2000 km^2^ worldwide where 829 CWR species related to 157 crops can be systematically conserved in situ. This analysis used both adaptive and pragmatic scenarios, adaptive resulting from individual CWR ELC analysis based on the native country range of each CWR clustered using non-collinear edaphic, geophysical and climatic sets of variables and pragmatic, which used the same approach but prioritizes sites containing protected areas (to maximize use of existing protection) and in a complementary fashion considers additional sites outside protected areas where there are CWR/adaptive scenarios combinations not identified within protected areas. The top 10 sites listed in Table 2 contain a combined total of 270 unique CWR (21.4% of assessed CWR) and 726 CWR/adaptive scenarios (5.1% of all genetic diversity), all contained within protected areas. Five of the top 10 sites are found in the Mediterranean basin and mainland Europe (in Spain, Greece, Italy, Austria and Turkey); additionally, two sites are in East Asia (in China and Myanmar), one in Southeast Asia (in Malaysia), one site in North America (in USA) and one site in South America (in Brazil). The protected areas that overlap the top 10 sites in Fig. 3 cover a range of regional and global designations including: Special Protection Areas (under the European Union’s Birds Directive)—Spain; Scenic areas (IUCN’s Management Category VI—China; Provincial/Regional Nature Reserves (IUCN V)—Italy; Sites of Community Importance (under the European Union’s Habitats Directive)—Greece; World Heritage sites—China; and, Indigenous Areas—Brazil. The top 10 sites outside protected areas listed in Table 3 complement the 100 sites in protected areas selected in the pragmatic scenario, and contain a combined total of 283 unique CWR (22.4% of total assessed CWR) and 836 CWR/adaptive scenario combinations (5.8% of total genetic diversity) from 106 crop gene pools; however, they only add 205 (16.3% of assessed CWR) species and 531 CWR/adaptive scenario combinations (3.7% of total genetic diversity) from 89 crop gene pools to the existing 100 sites in protected areas. Five of the sites listed in Table 3 are in the Fertile Crescent and Caucasus region; additionally, two are found in Central and North America, one in South America, one in Spain and one in Afghanistan. Effectively conserving the top 10 sites inside protected areas and the top 10 sites outside protected areas defined in the pragmatic scenario, would only require active management of ~2000 km^2^ globally and would protect 475 CWR species, and 1257 unique CWR/adaptive scenario combinations. Meanwhile, only 0.01% of the world’s total terrestrial area would be required to conserve the top 150 sites presented.

**Table 2:** Details of the top 10 CWR sites inside existing protected areas in the pragmatic implementation scenario

Site No.	Country	Location	No. CWR	No. unique CWR added to the reserve network	No. unique CWR/adaptive scenarios combinations added to the reserve network	Protected areas
1	Spain	Simat de la Valldigna, Benifairó de la Valldigna, Alzira, Tavernes de la Valldigna, Xeraco, Barx, Xeresa and Gandia, Valencia province	85	85	155	Montduver-Marjal de la Safor (Special Protection Area (Birds Directive), Regional); Serres del Montduver i Marxuquera (Site of Community Importance (Habitats Directive), Regional); Parpallo-Borrell (Nature Place (Local Interest), National); Serra de Corbera (Site of Community Importance (Habitats Directive), Regional)
2	Greece	Messinia and Laconia districts in the Peloponnese	58	15	91	Oros Taygetos (Site of Community Importance (Habitats Directive), Regional); Lagkada Trypis (Special Protection Area (Birds Directive), Regional)
3	China	Border of Xishan, Chenggong and Kunming counties in Kunming district	42	42	78	Dianchi (Scenic area, National, IUCN VI)
4	USA	Intersection of Skamania, Oregon and Hood River and Klickitat counties in Washington State	32	30	68	Wygant (State Natural Area, National, IUCN V)
5	Italy	Monreale, Corleone, Godrano, Piana Degli Albanesi and Marineo in Palermo province Sicily	71	10	61	Riserva naturale orientata Bosco della Ficuzza, Rocca Busambra, Bosco del Cappelliere e Gorgo del Drago (Regional/Provincial Nature Reserve, National, IUCN IV); Rocca Busambra e Rocche di Rao (Site of Community Importance (Habitats Directive), Regional)
6	Malaysia	Northern Ranau district, Sabah	37	33	61	Kinabalu (National Park and ASEAN Heritage Park, National, IUCN II)
7	Austria	North central Liezen district	30	6	59	Warscheneck-Gruppe (Landscape Protection Area, National, IUCN V); Ennstal von Ardning bis Pruggern (Landscape Protection Area, National, IUCN V); Ennstal zwischen Liezen und Niederstuttern (Special Protection Area (Birds Directive), Regional); Putterer See (Nature Reserve, National, IUCN IV); Schluchtwald der Gulling (Site of Community Importance (Habitats Directive), Regional)
8	China, Myanmar	Gongshan Derung and Nu county, China and Khawbude township, Myanmar	37	10	57	Three Parallel Rivers of Yunnan Protected Areas (World Heritage Site, International)
9	Turkey	Çamliyayla district, Mersin province	59	15	50	Cehennem Deresi Milli Parkı (National); Kadıncık Vadisi Milli Parkı (National)
10	Brazil	Intersection of Minaçu and Colinas do Sul districts, Goias	24	24	46	Ava-Canoeiro (Indigenous Area, National)

**Table 3:** Details of the top 10 CWR sites outside of protected areas in the pragmatic implementation scenario

Site No.	Country	Location	No. CWR	No. unique CWR added to the pragmatic reserve network	No. unique CWR/ASc combinations added to the pragmatic reserve network
1	Israel	North central HaZafon province	81	44	86
2	Armenia	Eastern Vayots Dzor province	57	30	75
3	USA	Warren, Page and Rappahannock counties, Virginia	30	23	58
4	Mexico	Dist. Yautepec and Dist. Miahuatlan, Oaxaca	31	26	51
5	Armenia/ Azerbaijan	South west Zangilan province, Azerbaijan and south east Syunik province, Armenia	73	20	50
6	Lebanon	Central Baalbek, Beqaa province	56	9	47
7	Afghanistan	Central eastern Dara-i-Pech, Kunar province	46	17	42
8	Israel	Northern Haifa district	79	3	42
9	Spain	Camaleño, Cillorigo de Liébana, Potes, Peñarrubia and Tresviso, Cantabria province	74	14	41
10	Bolivia	Yanacachi, Coripata and Coroico municipalities in La Paz	26	19	39

## Discussion

Our results identify 150 sites covering ~2000 km^2^ worldwide where 829 CWR species related to 157 crops can be systematically conserved in situ. One hundred of these sites are in current protected areas, so theoretically are under some form of existing conservation management, though that management is unlikely to be focused on the genetic conservation of the CWR populations present. The active in situ management of these 150 sites to maintain genetic diversity and their incorporation into a global CWR in situ conservation network would substantially improve the genetic breadth of CWR conservation and substantially contribute to global food security and poverty alleviation as required by the international conservation policy^8,12^.

The approach presented here prioritizes sites for conserving multiple CWR within existing protected areas to optimize overall cost/benefit. Protected area management can be adapted for CWR genetic in situ conservation, but the current global grid has gaps and 35 CWR occur solely outside protected areas, such as *Vicia hyaeniscyamus* found on the Lebanese/Syrian border. Actions that can contribute to in situ CWR conservation including establishing new protected areas or less formal in situ management sites is required. Unlike existing protected areas managed to preserve unique habitats or rare and threatened taxa, these would be genetic reserves, where the goal is to maintain or enhance the genetic diversity of the priority CWR, rather than species presence alone, irrespective of levels of intra specific genetic diversity. If establishing genetic reserves in existing protected areas, existing site/population management plans would require amendment and/or preparation to specifically address the requirement to maximize genetic diversity maintenance^19^. Other less formal in situ conservation approaches may also be employed, such as conservation easements, that establish voluntary agreements between conservation agencies and landowners restricting or limiting the development of a site^20,21^. This approach could aid the conservation of many CWR, particularly ruderal legumes and grasses, which are often found in disturbed habitats, such as roadside verges, and are often absent from conventional protected areas established to preserve pristine environments.

Our results indicate that the predicted impacts of climate change on CWR distributions vary widely among CWR, even within crop gene pools; therefore, it is important conservation strategies are adapted to individual taxon requirements. CWR such as *Zea perennis* and *Vicia hyaeniscyamus*, which are predicted to lose 100% of their current distribution by 2070, and *Arachis appressipila*, *Vigna keraudrenii* and *Manihot gabrielensis*, which are predicted to lose over 50% of their existing range by 2070, should be prioritized for ex situ conservation or even introduction to climate-proofed sites, such as those identified in our analysis. Further work is required to analyze the level of fragmentation CWR distributions are likely to face in the future as this would affect in situ conservation requirements and increase the need to plan for corridors or stepping stones between established reserves to promote migration and to maintain gene flow between populations. It should also be remembered, as recommended in the CWR genetic reserve quality standards^11^, that all CWR populations conserved in situ should be backed-up in ex situ collections, particularly given that until the global CWR in situ network is established, most CWR users will gain access to diversity via gene bank collections.

The methodology applied in this analysis is innovative, prioritizing sites found within existing protected areas, maximizing overall environmental (and thus potential genetic) diversity coverage and long-term site viability in view of climate change. Genetic diversity data per CWR in the analysis was estimated using environmental diversity as a proxy. Further study and experiments are required to test whether this approach is truly appropriate for such a wide range of taxa. It is noted that the increasing power and decreasing costs of direct measures of genetic diversity will be useful in refining conservation priorities^4^, but it seems unlikely such techniques will be practical in the near term for planning the conservation of as many as 829 CWR taxa throughout their global range. Incorporating actual genetic diversity and characterization data for individual occurrences into conservation planning on such a scale may be possible eventually, but in the meantime the approach taken here offers a practical methodology that can be applied widely in wild plant species conservation planning.

The occurrence dataset used in this analysis highlighted that many CWR are poorly represented in gene banks and herbaria and occurrence databases worldwide, with 164 CWR having no occurrence records at all and a further 470 CWR having fewer than 10; this reinforces the recommendations for increased CWR surveying and conservation^5^. The process of targeted global ex situ CWR collection which has begun^7^ will itself generate additional data for subsequent in situ CWR conservation planning, especially for rare and under-collected taxa. Some crop genepools are particularly under represented, such as citrus fruits, tea and tropical and sub-tropical fruit-bearing trees, possibly due to unresolved taxonomy in the case of *Citrus* or difficulty in collecting and conserving recalcitrant seeds in the case of tropical fruit trees. It may also be advisable in subsequent in situ gap analyses to weight target taxa, using relative gene pool level^22^ or IUCN Red List assessment^23^ of the CWR species to ensure those taxa easiest to cross with the crop or most threatened in the wild are prioritized during analysis.

Our results identify 150 sites where active in situ management might be most effective in maximizing CWR species and intra CWR genetic diversity inclusion and offer the highest chance of persistence under climate change. However, these are not the only sites worthy of active in situ management globally. Here, the basis of the analysis were the priority list of CWR taxa related to 167 global major crops^24^, the restriction to include only global major crops highlights Europe and the Middle East as the main centre of diversity, but the CWR diversity of minor crop of regional or local importance not included in this analysis also require active in situ maintenance. Therefore, analysis of these minor crop of regional or local importance and their CWR diversity is required to identify additional complementary to add to the 150 sites identified here.

Finally, we propose that the CWR in situ conservation sites, along with additional CWR sites of minor crop importance, can most efficiently be managed as a global CWR in situ conservation network that coordinates practical in situ conservation management, fostering stronger partnerships at national, regional and global levels, demonstrates benefits that directly support the ultimate custodians of agrobiodiversity, the local communities found in and around the included sites, and ultimately safeguard for perpetuity this critical resource for use either directly by farmers or by plant breeders and other scientists in crop improvement. Catalyzing better linkages between conservation and sustainable use of agrobiodiversity for the benefits of current and future generations is required^25^. However, the establishment of such a network is complex. For example, several of the 150 prioritized sites identified are located on country borders: Israel-Lebanon-Syria, Lebanon-Syria, Armenia-Azerbaijan and China-Myanmar; and two of the highest priority sites are in current conflict regions: Syria and Crimea. Therefore, although we highlight here a global matrix of 150 priority sites for in situ CWR conservation, the establishment of a global CWR in situ conservation network will need to be an incremental effort, starting pragmatically with a few sites/populations, and building upon those with time. Previous published work has identified global priority CWR taxa and broadly where they are located^24^, and how populations should be managed in situ^11,19^, from our analysis, we now know which combination of sites and CWR populations can form an effective network to maximize in situ CWR diversity conservation. It is now an urgent priority to identify existing and novel mechanisms to finance and govern the proposed network, the network that will provide a fundamental basis for ensuring our future food security. Failure to address this challenge is likely to have a devastating outcome for food production and agriculture, further the implications for rural people on low incomes in developing countries could be catastrophic, therefore action to prevent these outcomes is required immediately.

## Methods

### Selection of target CWR and occurrence data compilation

We selected the wild relatives of 167 crops of global importance for food security and farmer income generation, primarily based upon their ability to successfully cross with cultivated taxa and produce fertile offspring, and their known or potential use in plant breeding. We used the gene pool concept^26^ to determine the ability of CWR to successfully cross with crops and produce fertile offspring. Surrogate concepts were used where there was no hybridization data available^24^. Occurrence records for all target CWR species were obtained from a global CWR data set of ecogeographic records that is available online at http://www.gbif.org/dataset/07044577-bd82-4089-9f3a-f4a9d2170b2e^27^. CWR were recorded at the species level due to low availability of occurrence records for intraspecific levels. We removed non-target species, records of cultivated species, occurrence records outside their reported native range, records without coordinates and records with highly uncertain coordinates (i.e., more than 10 km uncertainty). Information of native species ranges were obtained from the GRIN-Global and the Harlan and de Wet portals. CWR taxonomic nomenclature in the occurrence record database was standardized to match GRIN nomenclature^28^.

### Current and future species distribution modelling

We chose the MaxEnt algorithm to model the potential distributions of CWR species due to its strong performance against other modelling algorithms, particularly when using small occurrence datasets, its ability to work with presence-only data, and its wide use in biodiversity conservation studies^29^. MaxEnt requires a background area and background points when absence data is not available. Moreover, defining the extent of the background area is key to reduce model overfitting and therefore to improve the performance of species distribution models produced with the algorithm^29^. We used the native geographic range of each CWR species to determine the background extent of each distribution model and produced ten thousand random background points within this. For environmental drivers, we selected an initial set of 27 edaphic, geophysical and climatic variables for input use in MaxEnt (Supplementary Information Table 1). We assessed whether high multicollinearity existed among the initial set of environmental drivers per CWR species by measuring the variance inflation factor (VIF). Variables with a VIF value ≥10 were removed from the final set of variables.

We projected each potential distribution model to baseline data for the period of 1960–1990 (Worldclim v.1.4; http://www.worldclim.org/). For climate change projections, we selected thirty global circulation models (GCMs) produced by the Coupled Model Intercomparison Project Phase 5 (CMIP5) (Supplementary Information Table 2). We chose a stringent emissions scenario (Representative Concentration Pathway 4.5 – RCP 4.5) for the period of 2060–2089 to regions highly likely to remain climatically stable, and thus adequate for long-term in situ conservation. All future climate data were obtained from http://ccafs-climate.org/. All environmental variables had a grid cell resolution of 5 km × 5 km at the equator. Only CWR species with ten or more unique occurrence records were considered for modelling, due to unreliability and poor performance of distribution models for species with smaller datasets^30^. Models were trained using a five-fold cross-validation technique to maximize the use of all occurrence records. Once trained, all models were first projected onto baseline variables. We assessed the performance of models produced by following standard adequacy criteria^30^. We produced binary distribution maps with the models that met the adequacy criteria, by applying the maximum training sensitivity plus specificity (MAXTRSS) logistic threshold, as this thresholding method has been found to consistently outperform other techniques^30^. For the CWR models that did not meet the adequacy criteria, or for species with fewer than 10 unique occurrence records, we opted to produce a 50 km circular buffer surrounding each individual georeferenced occurrence record to represent the potential distribution^31^. Only the current climate species models that met the validation criteria were projected onto each individual GCM. Then, we averaged all GCMs per species to produce a future ensemble model. The MAXTRSS threshold was again used to produce binary presence/absence distribution maps. We compared each future distribution against the current distribution model to obtain maps of geographical areas that are likely to remain climatically stable per species, and thus can be considered as suitable areas for long term in situ conservation of CWR.

### Assessment of potential genetic diversity

The genetic diversity of individual populations must be considered to ensure maximum coverage in protected areas, prevent genetic erosion in the wild, and ultimately to effectively conserve CWR in situ for future utilisation. Given the limited availability of molecular data for the CWR species selected in the study, we created an ELC map for each CWR, using species distribution models as a proxy to estimate potential genetic diversity^32^. We created an ELC map of the native country range of each CWR by clustering the non-collinear edaphic, geophysical and climatic sets of variables and combining each resulting cluster value to produce a map containing unique ELC categories (also referred to as adaptive scenarios). We then overlaid the ELC maps with the current potential CWR distributions to determine the breadth of adaptive scenarios per species, and thus potential genetic diversity.

### In situ gap analysis

We estimated the number of current and future distributions of CWR that are encompassed within established protected areas worldwide, to estimate the status of CWR species under passive in situ conservation, and the likely geographical losses under a stringent climate change scenario. For this, we obtained a comprehensive spatial dataset containing the geographical location of the world’s protected areas from www.protectedplanet.net (Downloaded 25/05/2016). Protected areas represented as point data were discarded and individual protected area polygons were transformed to produce a global presence/absence raster of protected areas. Then, we estimated the proportion of current and future potential distributions, including corresponding adaptive scenarios, within protected areas for each CWR and then summarized these per crop type. Finally, we compared the proportion of CWR area within protected areas and current adaptive scenarios that are likely to be lost due to climate change in the future.

### Prioritisation of areas for in situ conservation

We used Marxan, a widely used conservation planning software, to determine the most effective global reserve network to conserve all target CWR species and their individual adaptive scenarios. To run Marxan, we prepared the following input files: (1) The planning unit file was created by producing a terrestrial global grid (5 km × 5 km grid cell resolution at the equator), where each grid cell was assigned a unique identifier. We assigned a planning unit cost of 10 to grid cells that overlapped protected areas, and 50 to grid cells that did not overlap with protected areas. Lower cost units are prioritized as conservation units. Furthermore, we prioritized every planning unit grid cell that overlapped a protected area to ensure the final network maximised the inclusion of existing protected areas. (2) The species file was created by listing all CWR species and adaptive scenario combination as a concatenated string and assigning each a unique identifier. We set Marxan targets to achieve at least one of every CWR species and adaptive scenario combination in the final network. We calibrated the species penalty factor, which allows prioritization of biodiversity elements for selection in Marxan, using a standard technique^33^, resulting in a final species penalty factor of one for all species, to ensure equal chance of selection. (3) We created the planning unit versus species representation file by overlapping the distribution maps of each CWR/adaptive scenario combination with the planning unit file. We used the current distribution models for species with predicted full loss of current range due to climate change, or where a valid MaxEnt model was not produced. For species with a valid current and future MaxEnt distribution model, we used only the geographical distribution that is predicted to remain climatically stable in the future. (4) For the boundary file, we listed the numerical identifiers of the horizontal and vertical neighbours of each terrestrial planning unit cell. We added this file to improve the spatial clumping of selected sites, as it is often easier and more cost effective to conserve closely located sites rather than dispersed ones. (5) For the input parameters file, we set the Marxan scenario to perform 100 runs of 100,000,000 iterations. The boundary length modifier, which helps to produce spatially clumped network of potential conservation sites, was calibrated using the standard technique^33^ and set to 0.001.

The resulting Marxan solutions were then ranked by fewest number of planning units followed by lowest cost. We chose the top ranked solution as the most suitable overall solution. We further prioritized the planning units in the top ranked Marxan solution by using the complementarity ranking algorithm^34^ to maximise taxonomic and genetic diversity in the reserve network. This algorithm initially selects the site with highest richness count for CWR/adaptive scenario combination, and then chooses the second site that will be most complementary to the first, i.e. the site which will most increase the net number of CWR/adaptive scenario combinations. We used pragmatic scenario prioritizes sites containing protected areas (to maximise use of existing protection) and in a complementary fashion considers additional sites outside protected areas where there are CWR/adaptive scenarios combinations not identified within protected areas. It is advisable to identify site both inside and outside of existing protected areas because CWR taxa are commonly found in disturbed anthropogenic environments and less often found in the climax community often designated as conventional protected areas^35^. All CWR were given equal weighting in the algorithm and it was run until all CWR/adaptive scenario combinations were represented in the final solution at least once. The top 150 priority sites within the top ranked Marxan network were then mapped, the top 100 sites inside protected areas, along with the top 50 complementary sites outside protected areas.

### Reporting summary

Further information on experimental design is available in the Nature Research Reporting Summary linked to this article.

## Supplementary information

Description of Additional Supplementary Files

Supplementary Information

Supplementary Data 1

Reporting Summary

## Data Availability

Interactive maps displaying occurrence data coordinates, potential distribution models are available at http://www.cwrdiversity.org/ distribution-map/. Occurrence data used for this analysis are available at http:// www.cwrdiversity.org/checklist/cwr-occurrences.php. Further information on expert evaluations of the gap analysis are available at http://www.cwrdiversity.org/ expert-evaluation/. The entire dataset collated and used for the analysis is available from is available online at http://www.gbif.org/dataset/07044577-bd82-4089-9f3a-f4a9d2170b2e.
