# Supplementary material for: Modeling of crop wild relative species identifies areas globally for in situ conservation
Source: Commun Biol. 2019 Apr 23;2:136. doi: 10.1038/s42003-019-0372-z (PMC6478866; doi:10.1038/s42003-019-0372-z)
Supplement: Supplementary file 2 — Supplementary Information [file 42003_2019_372_MOESM2_ESM.pdf]

Description of Additional Supplementary Files

File Name: Supplementary Data 1
Description: Basic descriptive data for 1,425 CWR species.
