# Supplementary material for: Modeling of crop wild relative species identifies areas globally for in situ conservation
Source: Commun Biol. 2019 Apr 23;2:136. doi: 10.1038/s42003-019-0372-z (PMC6478866; doi:10.1038/s42003-019-0372-z)
Supplement: Supplementary file 3 — Supplementary Data 1 [file 42003_2019_372_MOESM3_ESM.pdf]

Supplementary Table **1 Variables used in species distribution and ecogeographic land characterization modelling.**

**Variable**	**Variable name**	**Units**	**Source**
1	Annual mean temperature	ºC	Worldclim
2	Mean diurnal temperature range	ºC	Worldclim
3	Isothermality	N/A	Worldclim
4	Temperature seasonality (standard deviation)	ºC	Worldclim
5	Maximum temperature of warmest month	ºC	Worldclim
6	Minimum temperature of coldest month	ºC	Worldclim
7	Temperature annual range	ºC	Worldclim
8	Mean temperature of wettest quarter	ºC	Worldclim
9	Mean temperature of driest quarter	ºC	Worldclim
10	Mean temperature of warmest quarter	ºC	Worldclim
11	Mean temperature of coldest quarter	ºC	Worldclim
12	Annual precipitation	mm	Worldclim
13	Precipitation of wettest month	mm	Worldclim
14	Precipitation of driest month	mm	Worldclim
15	Precipitation seasonality (coefficient of variation)	%	Worldclim
16	Precipitation of wettest quarter	mm	Worldclim
17	Precipitation of driest quarter	mm	Worldclim
18	Precipitation of warmest quarter	mm	Worldclim
19	Precipitation of coldest quarter	mm	Worldclim
20	Bulk density	kg/m^3	Soilgrids.org
21	Cation exchange capacity	cmol/kg	Soilgrids.org
22	Percent clay	%	Soilgrids.org
23	Organic carbon	g/kg	Soilgrids.org
24	pH in H_2O	pH	Soilgrids.org
25	Percent silt	%	Soilgrids.org
26	Percent sand	%	Soilgrids.org
27	Altitude	m	Worlclim

Supplementary Table **2 Variables used in climate change modelling.**

GCM	Institution
bcc_csm1_1_m	Beijing Climate Center, China Meteorological Administration
bcc_csm1_1	Beijing Climate Center, China Meteorological Administration
bnu_esm	Beijing Normal University
cccma_canesm2	Canadian Centre for Climate Modelling and Analysis
cesm1_bgc	National Center for Atmospheric Research
cesm1_cam5	National Center for Atmospheric Research
csiro_access1_0	CSIRO (Commonwealth Scientific and Industrial Research Organisation, Australia), and BOM (Bureau of Meteorology, Australia)
csiro_access1_3	CSIRO (Commonwealth Scientific and Industrial Research Organisation, Australia), and BOM (Bureau of Meteorology, Australia)
csiro_mk3_6_0	Commonwealth Scientific and Industrial Research Organisation in collaboration with the Queensland Climate Change Centre of Excellence
fio_esm	The First Institute of Oceanography, SOA, China
gfdl_cm3	National Oceanic and Atmospheric Administration
gfdl_esm2g	National Oceanic and Atmospheric Administration
gfdl_esm2m	National Oceanic and Atmospheric Administration
giss_e2_h_cc	NASA Goddard Institute for Space Studies
giss_e2_r_cc	NASA Goddard Institute for Space Studies
giss_e2_r	NASA Goddard Institute for Space Studies
inm_cm4	Institute for Numerical Mathematics
ipsl_cm5a_lr	Institut Pierre-Simon Laplace
ipsl_cm5a_mr	Institut Pierre-Simon Laplace
lasg_fgoals_g2	LASG, Institute of Atmospheric Physics, Chinese Academy of Sciences; and CESS, Tsinghua University
miroc_esm_chem	Japan Agency for Marine-Earth Science and Technology, Atmosphere and Ocean Research Institute (The University of Tokyo), and National Institute for Environmental Studies
miroc_esm	Japan Agency for Marine-Earth Science and Technology, Atmosphere and Ocean Research Institute (The University of Tokyo), and National Institute for Environmental Studies
miroc_miroc5	Atmosphere and Ocean Research Institute (The University of Tokyo), National Institute for Environmental Studies, and Japan Agency for Marine-Earth Science and Technology
mohc_hadgem2_cc	Met Office Hadley Centre (additional HadGEM2-ES realizations contributed by Instituto Nacional de Pesquisas Espaciais)
mohc_hadgem2_es	Met Office Hadley Centre (additional HadGEM2-ES realizations contributed by Instituto Nacional de Pesquisas Espaciais)
mpi_esm_lr	Max Planck Institute for Meteorology
mri_cgcm3	Meteorological Research Institute
ncar_ccsm4	National Center for Atmospheric Research
ncc_noresm1_m	Norwegian Climate Centre

nimr_hadgem2_ao	National Institute of Meteorological Research/Korea Meteorological Administration
